# On the Mechanism of Bioinspired Formation of Inorganic Oxides: Structural Evidence of the Electrostatic Nature of the Interaction between a Mononuclear Inorganic Precursor and Lysozyme

**DOI:** 10.3390/biom11010043

**Published:** 2020-12-30

**Authors:** Lucia Gigli, Enrico Ravera, Vito Calderone, Claudio Luchinat

**Affiliations:** 1 Magnetic Resonance Center (CERM)/Consorzio Interuniversitario Risonanze Magnetiche di Metalloproteine (CIRMMP), University of Florence, Sesto Fiorentino, 50019 Florence, Italy; gigli@cerm.unifi.it (L.G.); luchinat@cerm.unifi.it (C.L.); 2 Department of Chemistry “Ugo Schiff”, University of Florence, Sesto Fiorentino, 50019 Florence, Italy; 3 CNR ICCOM, Sesto Fiorentino, 50019 Florence, Italy

**Keywords:** lysozyme, titanium, biomineralization, inorganic oxides

## Abstract

Nature has evolved several molecular machineries to promote the formation at physiological conditions of inorganic materials, which would otherwise be formed in extreme conditions. The molecular determinants of this process have been established over the last decade, identifying a strong role of electrostatics in the first steps of the precipitation. However, no conclusive, structure-based evidence has been provided so far. In this manuscript, we test the binding of lysozyme with silica and titania potential precursors. In contrast with the absence of structural information about the interaction with the silica precursor, we observe the interaction with a mononuclear titanium(IV) species, which is found to occur in a region rich of positive charges.

## 1. Introduction

The formation of inorganic oxides usually proceeds under harsh chemical conditions of extreme pH and high temperatures [1,2]. However, biological systems have evolved a vast array of machineries to promote the formation of inorganic oxides under conditions compatible with life (neutral pH and room temperature) [3,4]. Some of them are specific for one oxide (e.g., silica) but show competence towards formation of other oxides as well [5,6]. In 2006, Luckarift et al. [7] demonstrated that lysozyme is able to template the formation of silica and titania nanoparticles from the aqueous solutions of their precursors. From this seminal paper, other applications demonstrating the tunability of this preparation method have appeared in the literature [8], opening “de facto” a totally new field in nanotechnology (see references [9,10,11,12,13,14] and references therein). The potential applications of these composites are many, from cosmetics [15] to photocatalysis applications [6,8].

Proteins and biomolecules that template the formation of silica and titania tend to be polycationic [16,17,18,19,20,21,22,23,24,25,26,27,28,29,30], and this appears to point towards a role for the electrostatics of the interaction of the protein with the precursors. However, no structural evidence has been provided so far. In this manuscript, we explore the interaction of silica and titania precursor with lysozyme using X-ray crystallography.

## 2. Materials and Methods

### 2.1. Crystallization, Data Collection, and Structure Solution

Hen-Egg White Lysozyme (HEWL) has been purchased from Sigma-Aldrich (Milan, Italy) and used without any further purification.

Crystals of HEWL were obtained in hanging drop by adding an aliquot of 2 µL of protein solution (0.5 mol/dm^3^ Tris-HCl, 8 mg/mL HEWL, pH 8.5) to 2 µL of reservoir buffer (0.5 mol/dm^3^ Tris-HCl, 0.7 mol/dm^3^ NaCl, pH 8.5) and stored at 4 °C. The protein concentration in the sample was 8 mg/mL.

The crystals were afterwards soaked in tetraoxosilicic(IV) acid and titanium(IV) bis(ammonium lactato)dihydroxide (TiBALDH) solution, in different concentrations (20–100 mmol/dm^3^ in buffer at pH 8.5 for both precursors and 1.7 mol/dm^3^ for TiBALDH only) for 5 days. The dataset was collected in-house, using a BRUKER (Milan, Italy) D8 Venture diffractometer equipped with a PHOTON II detector, at 100 K; the crystal used for data collection were cryo-cooled using 25% ethylene glycol in the mother liquor. The crystal is diffracted up to 1.8 Å resolution; it belongs to space group P4_3_2_1_2 with one molecule in the asymmetric unit, a solvent content of about 50%, and a mosaicity of 0.3°. The data were processed keeping Friedel mates separate using the program XDS [31], reduced and scaled using XSCALE [31], and amplitudes were calculated using XDSCONV [31]. The structure was solved using the molecular replacement technique and showed the presence of one molecule in the asymmetric unit; the successful model used was 2W1X. The successful orientation and translation of the molecule within the crystallographic unit cell was determined with MOLREP [32]. The refinement was carried out using PHENIX [33], applying TLS restraints and using anisotropic B-factors for Na, Cl, and Ti only. In between the refinement cycles, the model was subjected to manual rebuilding using COOT [34]. Water molecules have been added using the standard procedures within the ARP/WARP [35] suite. The quality of the refined structure was assessed using the program MOLPROBITY [36]. Data processing and refinement statistics are shown in Table 1. Coordinates and structure factors have been deposited at the PDB under the accession code 7A70.

(1) $$†R_{merge}=\frac{\sum_{hkl}\sum_{i}\left|I_{i}\left(hkl\right)\;-\;\langle I\left(hkl\right)\rangle \right|\;}{\sum_{hkl}\sum_{i}I_{i}\left(hkl\right)},$$

where *I_i_*(*hkl*) is the mean intensity of the *i*-th observation of symmetry-related reflections *hkl*.

(2) $$‡R=\frac{\sum_{hkl}\left|\left|F_{obs}\right|\;-\;\left|F_{calc}\right|\right|}{\sum_{hkl}\left|F_{obs}\right|},$$

where *F_calc_* is the calculated protein structure factor from the atomic model (*R_free_* was calculated with a randomly selected 5% of the reflections).

### 2.2. DFT Calculations

The structure of Ti(OH)_4_ was taken from the present structure and subjected to refinement at the DFT level of theory, with the B3LYP functional [37,38,39,40], using Ahlrichs polarized basis set def2-TZVP [41,42] and Grimme’s dispersion correction D3 [43,44]. The resolution of identity approximation [45,46] was employed with auxiliary basis set def2-TZVP/J in order to speed up the calculations. CPCM implicit solvent (water) was used [47]. All calculations were carried out using the ORCA 4.2.1 quantum chemistry package [48,49].

## 3. Results and Discussion

The aim of the present study is to find structural evidence of interaction between inorganic precursors and HEWL in the initial steps of bioinspired oxide formation and is framed on a wider research that aims at elucidating the structure–activity relations in bioinspired preparation of inorganic oxides [27,29,50,51,52].

We performed an NMR titration with the precursor under the conditions that are used for the bioinspired mineralization, as described in reference [7].

For tetraoxosilicic(IV) acid, the only apparent perturbation at the highest precursor concentration is a 5% decrease in the signal intensity, with no detectable shift alteration (Figure S1). The superposition of the spectra in the absence and presence of tetraoxosilicic(IV) acid shows no significant differences.

For TiBALDH, shifts in the signals of residues 101–110 can be observed (Figure S2), but this area is known to be susceptible to variations in response to minor changes in the experimental conditions such as pH, ionic strength, etc. [53,54] and are often unresolved in some crystal forms as well [55,56]. Given that the pH has decreased from 8.5 to 5.1 during the addition, these results cannot be interpreted reliably.

HEWL is not only the prototypical templating molecule in bioinspired mineralization of silica and titania [7] but also particularly suitable for an X-ray crystallographic characterization, i.e., it easily crystallizes in a variety of conditions. (there are as many as 871 HEWL entries in the PDB as of September 2020, with 26 deposited in 2020) and because the quality of the crystals is usually high enough to allow for high-resolution in-house data collections.

The crystallization conditions were chosen to respect the pH at which the polymerization occurs (8.5) and to minimize the number of additives.

Soaking with the tetraoxosilicic(IV) acid solution (buffered at pH 8.5) resulted in fast disruption of the crystals, proportional to the concentration of tetraoxosilicic(IV) acid added to the drop. At the concentrations that are compatible with the crystals, no conclusive evidence of the presence of silicon species in the crystals can be found. The situation is markedly different for the titania precursor, as higher concentrations of the precursor do not disrupt the crystal order.

TiBALDH as a precursor for bioinspired titania synthesis is routinely employed because it is reported to be water-soluble and stable at neutral pH and ambient conditions [5,6,7,57,58,59,60]. TiBALDH solutions contain several species that are in equilibrium among themselves and with TiO_2_, including mononuclear species. At neutral or slightly basic pH, mononuclear hydrated titanium species also include Ti(OH)_4_ [5,61,62,63,64].

Soaking with buffered solutions at moderate concentrations of TiBALDH (20–100 mmol/dm^3^) did not cause alterations in the resulted crystal structures that indeed did not reveal a clear presence of titanium species. This was not fully expected because there is a report of a clear binding of a titanium species to the same crystal form of lysozyme (PDB entry: 6G5C) [65]. However, a very careful inspection of this entry casts doubts in terms of the presence of titanium (a putative TiO_2_ species in this case) for at least three reasons: the first one is that the supposed position of titanium perfectly corresponds to the position of a chloride ion in all lysozyme structure belonging to the same space group. The second one is the shape of the 2F_o_-F_c_ density of the putative titanium that is perfectly spherical and fits way better with the chloride ion. The third one is that mononuclear TiO_2_ species do not exist as such in solution [61,66].

For this reason, the last attempt was to perform a soaking with 2 µL of 50% *w*/*w* TiBALDH solution (1.69 mol/dm^3^, pH 8, subject to change upon the hydrolysis of the components). At variance with tetraoxosilicic(IV) acid and lower concentrations of TiBALDH, this did not cause a complete disruption of the crystals but slightly decreased the maximum resolution reached by these crystals. The data obtained with this last sample had a maximum resolution achievable of 1.8 Å instead of the typical 1.4–1.5 Å in the same diffractometer. Nevertheless, despite the lower resolution, the quality of the data remains very good and this has indeed allowed what is presented hereafter.

Considering the crystallization conditions, the only atoms which could have anomalous effect at the diffractometer wavelength are sulfur, chlorine, and titanium, with the last being the one with the largest expected value. For this reason, the data were processed keeping the Friedel mates separate with the hope to have some useful hints through anomalous difference maps.

In fact, these maps contoured at 3.0 σ value show clear peaks for all sulfur atoms and for the expected chloride atoms but, interestingly, they also show a peak in an unexpected position, which we interpreted as the hydrolyzed titanium compound in the form of Ti(OH)_4_.

**Figure 1:**
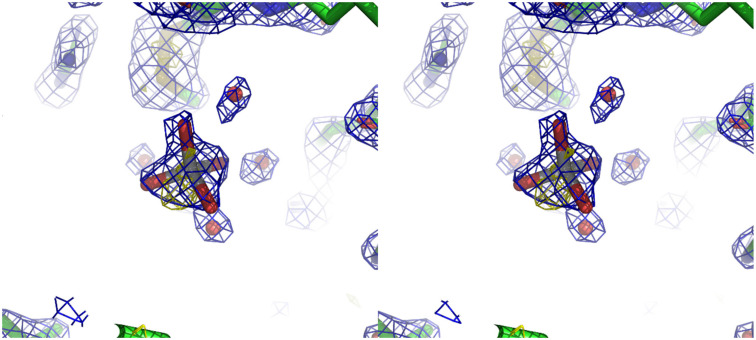
Cross-eye stereo representation of the electron density detail of the mononuclear titanium species and its environment. Water molecules are represented as red spheres. In blue is the 2F_o_-F_c_ contoured at 1.2 σ level, and in yellow is the anomalous difference map contoured at 3.0 σ value.

There are several reasons that led us to assess the presence of titanium with an occupancy of about 0.7:

(i) the presence of an anomalous signal is slightly higher than those attributed to sulfurs and chlorides;
(ii) the shape of the 2F_o_-F_c_ density is clearly not spherical as one would expect for chloride ion but rather tetrahedral as shown in Figure 1, and this becomes even more apparent when slightly lowering its contour value;
(iii) the Ti-O distances refine well at about 1.9 Å, which is in agreement with the theoretical value expected for such bond;
(iv) the F_o_-F_c_ density in that position is absent at ±3.0 σ contour;
(v) the B-factor values for the refined atoms of the titanium moiety have values coherent with those of the other labile or loosely bound atoms in the structure;
(vi) the comparison with several other lysozyme structures with the same space group shows that no density is present in the position that we attributed to the mononuclear titanium species.

With this result at hand, we have re-examined the data collected on the crystal soaked with 100 mmol/dm^3^ TiBALDH solution. These data show, ex post, a weaker but clear anomalous signal in the same position where we have identified titanium in the crystal soaked with the pure ligand.

The position and the binding mode of the ligand suggests that its presence is likely to be an artifact due to the crystal packing and to its very high concentration. In fact, the titanium compound is placed in the region between two symmetry-related molecules and has no direct chemical interaction with any of the protein atoms. It is held in place by hydrogen bond interactions with two well-defined water molecules that are, in turn, in close interaction with the protein (one with the backbone amide of Glu7 and the other one with the sidechain of Arg14 of a symmetry mate). The pattern is completed by two more interactions with two more labile water molecules, one of which interacts with the backbone amide of Cys6.

However, even if this interaction mode would be impossible if the protein was free in solution, it allows us to observe experimentally that the titanium species is mainly surrounded by positively charged residues. An electrochemical analysis of mononuclear titanium(IV) species present in aqueous solution as a function of the pH indicates that the species Ti(OH)_4_ is prevailing at the working pH [61], and a simple DFT calculation in implicit solvent indicates that the oxygen atoms can have up to 0.87 e^-^ partial charge, which can easily account for a preference for forming hydrogen bonds as acceptor.

**Figure 2:**
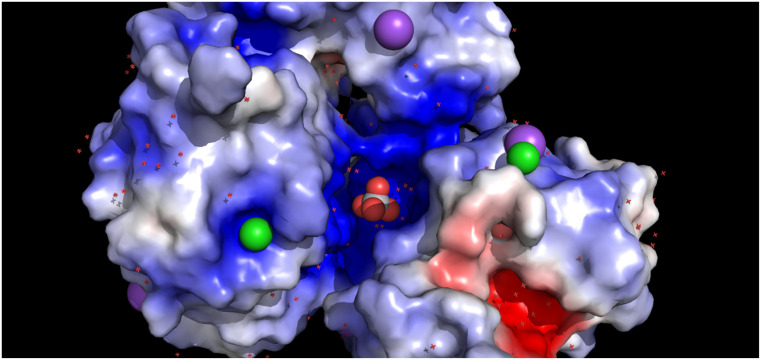
Surface representation (calculated with PyMOL APBS plugin) of the environment in which the titanium species is found, colored by electrostatic potential showing chloride ions in green and sodium in purple.

A plausible explanation for this fact could be the very high concentration of titanium(IV). This could cause the diffusion of Ti(OH)_4_ at a higher rate in the solvent channels, until the complex reaches a narrower channel where it forcibly stopped because of steric hindrance and charge accumulation (as illustrated in Figure 2).

At this point, the ligand can establish favorable interactions with water molecules that are kept in position by strong interactions with the charged amino acid residues around. This could, in turn, explain why the interactions that it establishes are not specifically targeted to some protein residues.

**Figure 3:**
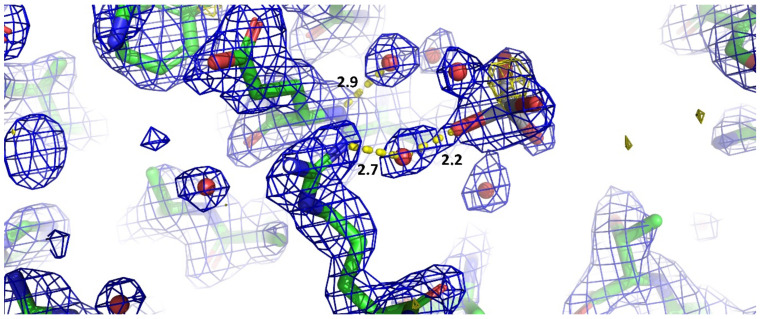
Detail of the two hydrogen bonds stabilizing the position of the titanium species through two water molecules represented as red spheres. Distances are expressed in Å. The 2FoFc map is contoured at 1.0 σ level.

It is also possible to speculate that the strongly positive environment in which the mononuclear titanium(IV) species is found makes it easier a further proton dissociation from the hydroxyl groups. This speculation could be supported by the interaction with a water molecule (wat11, Figure 3), which, in fact, establishes strong hydrogen bonds with Arg14 and with one of the hydroxyl groups of the titanium species. Since Arg14 is positively charged, then it must be the negative side of wat11 dipole to be responsible for the interaction with arginine, whereas the positive side of the water dipole interacts with the oxygen of one hydroxyl groups of the titanium, where the oxygens have a rather large negative partial charge.

## 4. Conclusions

The interest in bioinspired materials preparation notwithstanding, the interaction between the precursor of inorganic oxides and polycationic biological macromolecules has resulted elusive so far.

In this work, we tested the binding of lysozyme with silica and titania potential precursors. The silica precursor does not show any interaction with lysozyme in NMR experiments and causes the disruption of the protein crystals impeding X-ray diffraction studies. The situation is markedly different for the titania precursor. NMR spectra provide, in fact, detectable shifts but they cannot assess the binding beyond any reasonable doubt and, on the other side, the addition of TiBALDH to the crystals does not disrupt them, allowing for good resolution data collection. In this manuscript, we thus provide the first structure-based experimental evidence that among the possible mononuclear titanium species considered in the literature [62], Ti(OH)_4_ does interact with lysozyme before precipitation starts and that the interaction is electrostatic in nature. The interaction appears, in line with expectation, to be directed in an area where several arginine residues are present but, unexpectedly, appears to be mediated by an intervening water molecule; in fact, during its diffusion into the crystal, the titanium(IV) species happens to be trapped in a solvent channel created by symmetry mate molecules, because of steric hindrance and charge accumulation in the channel. This interaction is likely not occurring *as such* in solution under the usually applied reaction conditions. Nevertheless, our observation is a clear structure-based evidence of the existence of electrostatic interactions between the protein and one mononuclear titanium(IV) species and can be a proxy of those interactions that drive the initial steps of the oxide formation. We thus expect that this result will be a relevant starting point for detailed (e.g., computational [67,68,69]) studies of the structural basis of the bioinspired titania precipitation.

## Figures and Tables

**Table 1:** Data collection and refinement statistics.

Diffraction Source	BRUKER D8 Venture
Wavelength (Å)	1.541
Temperature (K)	100
Detector	PHOTON II
Crystal-detector distance (mm)	50
Oscillation range (°)	0.5
Total rotation range (°)	360
Exposure time/image (s)	30
Space group	P4_3_2_1_2
a, b, c (Å)	78.1, 78.1, 37.3
Mosaicity (°)	0.3
Resolution range (Å)	50.00–1.80 (1.91–1.80)
Total reflections	274,135 (24,437)
Unique reflections	20,052 (2909)
Completeness (%)	97.8 (89)
CC1/2	99.9 (45.1)
I/(σI)	16.1 (1.7)
*R_merge_* †	0.12 (0.91)
Wilson B factor (Å^2^)	29.9
*R_cryst_/R_free_* ‡ (%)	19.6/25.6
Protein atoms	1001
Water molecules	82
Ligand atoms	13
RMSD bond lengths (Å)	0.010
RMSD bond angles (º)	1.950

## Supplementary Materials

The following are available online at https://www.mdpi.com/2218-273X/11/1/43/s1, Figure S1: ^1^H NMR spectra of lysozyme titration with tetraoxosilicic(IV) acid, Figure S2: ^1^H NMR spectra of lysozyme titration with TiBALDH. References [70,71] are refered to in Supplementary Materials.
