# Supplementary material for: On the Mechanism of Bioinspired Formation of Inorganic Oxides: Structural Evidence of the Electrostatic Nature of the Interaction between a Mononuclear Inorganic Precursor and Lysozyme
Source: Biomolecules. 2020 Dec 30;11(1):43. doi: 10.3390/biom11010043 (PMC7823628; doi:10.3390/biom11010043)
Supplement: Supplementary file 1 [file biomolecules-11-00043-s001.pdf]

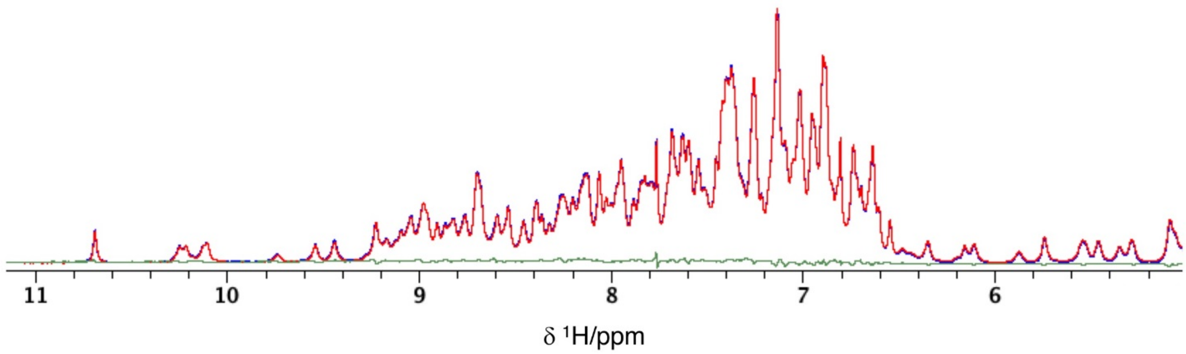

Figure S1. ^{1}H NMR spectra of lysozyme 10 mg/mL (red), lysozyme 10 mg/mL and silicic acid 100 mmol/dm^{3} (blue), and their difference (green).

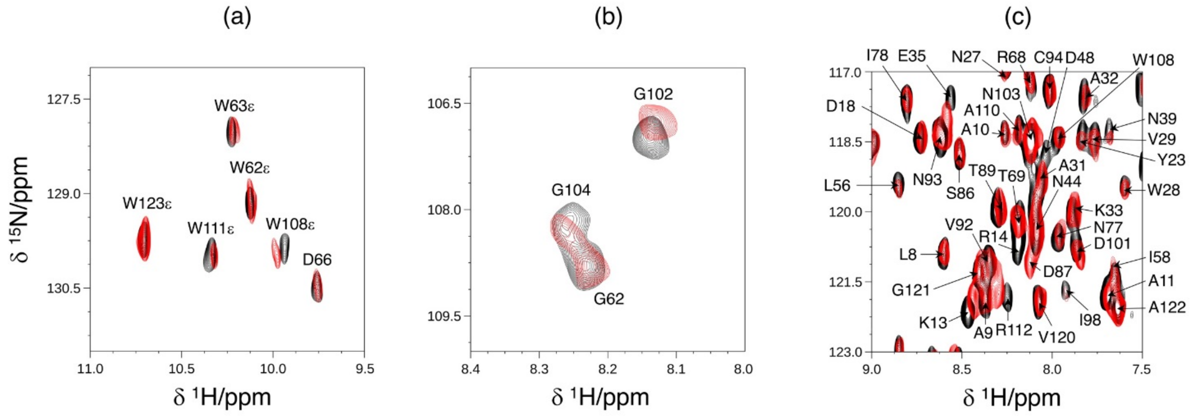

Figure S2. Enlargements of regions of the ^{1}H-^{15}N HSQC spectra of lysozyme 100 mg/mL (black), lysozyme 100 mg/mL and TiBALDH 100 mmol/dm^{3} (red). The assignments are taken from [1,2].

References

1. Buck, M.; Boyd, J.; Redfield, C.; MacKenzie, D.A.; Jeenes, D.J.; Archer, D.B. and Dobson, C.M. Structural Determinants of Protein Dynamics: Analysis of 15N NMR Relaxation Measurements for Main-Chain and Side-Chain Nuclei of Hen Egg White Lysozyme. *Biochemistry* **1995,** *34*, 4041–4055.
2. Kamatari, Y.O.; Yamada, H.; Akasaka, K.; Jones, J.A.; Dobson, C.M. and Smith L.J. Response of Native and Denatured Hen Lysozyme to High Pressure Studied by 15N/1H NMR Spectroscopy. *Eur. J. Biochem.* **2001,** *268*, 1782–1793.
